# Changes in Food Consumption Patterns After the Onset of the COVID-19 Pandemic Based on Age and Sex

**DOI:** 10.3390/nu17111754

**Published:** 2025-05-22

**Authors:** Lillie Monroe-Lord, Azam Ardakani, Ricardo Brown, Elmira Asongwed, Xuejing Duan, Tia Jeffery, Phronie Jackson

**Affiliations:** 1 Department of Health, Nursing and Nutrition, College of Agriculture, Urban Sustainability and Environmental Sciences, University of the District of Columbia, Washington, DC 20008, USA; azam.ardakani@udc.edu (A.A.);; 2 Department of Data Analytics, McDaniel College, Westminster, MD 21157, USA

**Keywords:** COVID-19, dietary habit, sex, age, nutritional risk

## Abstract

**Background**: The global outbreak of Coronavirus Disease 2019 (COVID-19) has significantly altered everyday routines, even influencing dietary behaviors and food choices. This study aimed to investigate the impact of the COVID-19 pandemic on changes in the consumption of various food sources and to examine the likelihood of nutritional vulnerability while exploring potential age and sex differences. **Methods**: This study employed a cross-sectional design through an online survey using the Qualtrics platform. Participants’ nutritional risk was assessed both before and after the onset of the COVID-19 pandemic (retrospectively) using the Dietary Screening Tool. This study involved a large sample of 10,050 participants aged between 40 and 100 years. The main outcome measures included changes in food consumption after the onset of the COVID-19 pandemic (from 9 August to 15 September 2020) and the likelihood of being classified as “nutritionally vulnerable” across different age and sex groups. Paired-samples *t*-tests were used to compare dietary changes before and after the onset of the COVID-19 pandemic, chi-square tests were used to explore categorical differences, and binary logistic regression was used to assess the likelihood of nutritional vulnerability. **Results**: The analysis revealed significant sex- and age-related differences in food consumption patterns after the onset of the pandemic. Men had a 30% greater likelihood of decreased dairy and processed meat consumption than women after the onset of the COVID-19 pandemic. Significant reductions in the consumption of processed meats (130%), vegetables (96%), lean protein (33%), and dairy (11%) were observed among individuals aged 40–60 years compared to those aged 81–100 years. The 61–80-year age group had a greater reduction in dairy consumption (21%) than the 81–100-year age group. Furthermore, women exhibited 17% greater odds of being classified as “nutritionally vulnerable” after the onset of the COVID-19 pandemic. However, age did not emerge as a significant predictor of nutritional vulnerability. **Conclusions**: The findings of this study can inform public health practitioners when developing interventions for improving dietary habits during and after pandemics, especially among vulnerable populations.

## 1. Background

The COVID-19 pandemic profoundly affected the global food supply chain, food accessibility, and dietary habits of individuals worldwide. The implementation of lockdowns and pandemic-related restrictions significantly altered food consumption patterns. While some studies reported a reduction in food consumption and the adoption of healthier eating practices during the COVID-19 lockdown, others revealed an increase in snacking, meal frequency, and unhealthy food choices [1,2,3,4,5,6,7,8,9,10,11,12]. These observations suggest that the lockdown had both positive and negative effects on dietary habits, which may have both immediate and long-term health implications [13].

Age is also a crucial sociodemographic factor that influences the food behavior of individuals [14]. People belonging to different age groups had varied food consumption patterns during the pandemic [15]. Due to the natural decline in their immune system and the greater prevalence of underlying health conditions in some individuals within this group, older adults are generally at greater risk of severe health issues and death from COVID-19 [16]. COVID-19 disproportionately impacts older adults; individuals aged 60 years and above, especially those over the age of 80 years, are at a significantly greater risk of severe outcomes and mortality. The above studies synthesized early data on the COVID-19 case fatality ratio (CFR) from China, Italy, and the United States, illustrating a stark age-related increase. The CFR is markedly lower in patients aged 40 years or younger—below 0.4% in both China and Italy and up to 3.3% in the United States. In stark contrast, for individuals aged 80 and above, the CFR increased to 14.8% in China, 20.2% in Italy, and 25.9% in the United States, underscoring the heightened vulnerability of older adults to severe outcomes [17,18,19,20]. Although several studies have examined the impact of COVID-19 on the health status of different age groups, there remains a significant gap in research on how dietary patterns have changed during the pandemic among individuals of different ages, especially middle-aged and older adults, who are more vulnerable than their younger counterparts.

The impact of the pandemic varied between women and men across different global regions. Several studies indicated that women faced more severe consequences from COVID-19 than men did in several aspects [21,22,23]. For instance, women disproportionately experienced challenges in food security and nutrition, restricted access to healthcare, diminished economic opportunities, and increased gender-based violence. These factors likely contributed to heightened food insecurity among women [24,25]. Furthermore, during periods of stress, women commonly engage in emotional eating, consuming more food than men, and show a tendency to stockpile food from grocery stores [26,27]. Interestingly, after engaging in emotional eating, women are more likely than men to experience guilt [28]. Studies on the snacking behavior of women during these challenging times have shown mixed results; some have reported increased snacking, potentially due to increased anxiety, while others have pointed to healthier eating habits compared with men [26]. In addition, some evidence suggests a notable increase in women’s consumption of fruits and vegetables compared with men’s consumption during this period. The consumption of animal protein decreased among both men and women during the pandemic. This shift is likely linked to diminished incomes, the shutdown of meat-processing facilities causing an increase in meat prices, and widespread evidence of animal-borne virus transmission [13,29,30]. Thus, it is crucial to monitor changes in people’s dietary habits after the onset of the COVID-19 pandemic due to gender disparities.

The gender-transformative framework for nutrition (GTFN) is a theoretical approach that recognizes how gender inequalities affect food security and nutritional outcomes [31,32,33] and is associated with COVID-19 in various ways. For instance, during a pandemic, women may face additional challenges in accessing nutritious food due to economic and social factors [24]. Therefore, interventions aimed at improving nutritional outcomes should consider sex-based barriers and inequalities and address the root causes that perpetuate these inequalities. Overall, the GTFN can provide a useful lens for understanding the sex-specific impacts of COVID-19 on nutrition and developing interventions that prioritize sex equity. Another possible theoretical framework for understanding the relationships among COVID-19, dietary changes, and age is the life course perspective (LCP). This framework emphasizes that an individual’s health behaviors and outcomes are influenced by multiple factors that occur over the course of life, including early life experiences, transitions and events, and cumulative advantages and disadvantages [34]. The LCP could provide a useful framework for understanding the complex and dynamic interplay between age, COVID-19, and dietary changes.

Many studies on dietary habits during the COVID-19 pandemic predominantly focused on European populations and were conducted at the early stage of lockdowns [3,6,9,10,12,25]. Recognizing this gap, the current study aimed to provide a comprehensive understanding of the impact of the pandemic on dietary habits in the later stages, particularly in the United States. Unlike previous research, this study encompasses a detailed analysis of all MyPlate food items, which were notably absent in earlier studies. *MyPlate* is a visual guide developed by the United States Department of Agriculture (USDA) to promote healthy eating habits. It divides a standard plate into five key food groups: vegetables, grains, protein, fruits, and dairy, reflecting the core components of a balanced diet [35]. By exploring the eating patterns among individuals of various ages and sex demographics in the U.S., this research aims to highlight how these factors influenced dietary patterns after the onset of the COVID-19 pandemic. Understanding these dynamics is vital for developing targeted public health interventions, especially for vulnerable populations, as they navigate the unique nutritional challenges posed by the pandemic. This insight is essential for informing future nutritional guidelines and policies, ensuring that they are tailored to the needs of diverse groups during such global health crises. Given emerging evidence that dietary behaviors during health crises differ by demographic factors such as age and sex [3,13,24,26], this study aimed to investigate (1) how the COVID-19 pandemic influenced the consumption of MyPlate food groups and FSS items among adults aged 40 and older, (2) whether these dietary changes significantly differed by sex and age group, and (3) whether sex or age predicted a higher likelihood of becoming nutritionally vulnerable during the pandemic.

## 2. Methods

### 2.1. Design, Participants, and Procedures

This cross-sectional study aimed to explore the impact of COVID-19 on the dietary habits of vulnerable urban populations. The study protocol was approved by the institutional review board of the University of the District of Columbia (IRB # 138067-4), and all participants provided written informed consent. A total of 10,050 participants, aged 40–100 years, were recruited between 9 August and 15 September 2020 through the Qualtrics online survey platform [36]. The inclusion criteria required participants to be between the ages of 40 and 100 years, reside in urban areas of the United States (defined by the U.S. Census Bureau as cities with populations over 50,000) [37], and have the ability to complete an online survey, which implied access to the internet and basic digital literacy. Participants were specifically selected due to their heightened vulnerability to severe COVID-19 symptoms or complications. Individuals under the age of 40, living in rural areas, or residing outside the U.S. were excluded from this study. This study focused on urban populations due to evidence that rural adults generally exhibit poorer diet quality compared to their urban counterparts, including lower intake of protein and fiber and higher intake of added sugars [38]. Rural residents also experience higher rates of obesity and physical inactivity, and even after adjusting for individual behaviors, they remain at greater risk—suggesting the influence of ‘obesogenic’ environments in rural areas [39]. Additionally, responses with significant missing data or those failing attention checks embedded in this survey were also excluded to ensure data quality and validity. These measures ensured the recruitment of a diverse and representative sample relevant to this study’s objectives.

### 2.2. Demographic Status

Participants’ data based on sex (male, female), race (White, Asian, Black, Hispanic), and ethnicity (Hispanic, non-Hispanic) were collected. In addition to race and ethnicity, a minority status construct was defined as “yes” if the race was anything other than White (non-Hispanic) and “no” otherwise. The participants were classified into three groups based on age: the 40–60-year age group (“40–60”), the 61–80-year age group (“61–80”), and the 81–100-year age group (“81–100”). This classification allowed for comparisons between middle-aged, early senior, and advanced senior populations in line with public health standards.

### 2.3. Dietary Assessment

The participants’ nutritional risk was evaluated using the 25-item short-form Dietary Screening Tool (DST), which was developed and validated for use in older and middle-aged populations [40,41]. The DST is specifically designed to gather detailed information about the frequency and types of food consumed by individuals. Each question within the DST is associated with specific food groups, color-coded for ease of reference. The DST questions have varying response options, with each assigned a score, and the total score ranges from 0 to 100. A higher score indicates a healthier diet, and the questions assess various food groups and dietary habits. For example, the frequency of fruit consumption as a snack is scored from 0 (never) to 5 (3 or more times a week), while the intake of processed meats such as cold cuts and bacon ranges from 5 (never or less than once a week) to 0 (3 or more times a week), reflecting the dietary recommendations for reduced processed meat intake. The DST was retrospectively administered twice, before and after the COVID-19 pandemic, to measure the changes in the participants’ nutritional risk over time [42]. The participants were categorized into three groups based on changes in their food intake after the onset of the COVID-19 pandemic: (1) decreased consumption, (2) no change in consumption, and (3) increased consumption.

To evaluate the dietary habits of the sample, the DST questions were categorized based on MyPlate items, including fruits, vegetables, grains, dairy, proteins (divided into lean protein and processed meat), and fat, sugar, and sweet (FSS) items [40,41,43,44]. The total DST score was subsequently calculated for each participant, and based on the scores, the sample was categorized into three groups: “at risk” (DST score < 60), “possible risk” (DST score between 60 and 75), and “not at risk” (DST score > 75). This categorization provided insight into the nutritional risk of the participants, thus allowing the analysis of the level of dietary risk [40,41,45].

### 2.4. Software and Statistical Tests

SAS 9.4 software (SAS Institute, Cary, NC, USA) was used for data analysis. Descriptive statistics are reported as the mean and standard deviation (SD) for continuous variables and as the frequency and percentage for categorical variables. Paired-samples *t*-tests were used to examine the differences in food items before and after the COVID-19 pandemic. Missing data were handled using listwise deletion, excluding participants with incomplete key responses. Approximately 5.71% of participants had missing data. The chi-square test was used to explore whether there was a significant association between two categorical variables, including changes in food consumption and nutritional risk status by sex and age. Furthermore, we constructed binary logistic regression models with MyPlate food items as the response variable, and Fisher’s scoring optimization technique was used for the model. The models predicted the probability of decreased consumption of each food item after the onset of the COVID-19 pandemic while considering explanatory variables, including sex, race, age, income, and education. In addition, odds ratio (OR) estimates were provided. Another binary logistic regression model that predicted the probability of belonging to a group with higher nutritional risk based on several predictor variables was used. The response variable was binary with two levels: those who did not have nutritional risk and those with nutritional risk (nutritionally vulnerable). Nutritional vulnerability was assessed by measuring whether the participants were more “at risk” after the onset of the COVID-19 pandemic than they were before. Therefore, people who were at risk both before and after the onset of the COVID-19 pandemic were not considered to constitute a “nutritionally vulnerable” group. However, people who went from “possible risk” to “at risk” were considered to be in the “nutritionally vulnerable” group after the onset of the COVID-19 pandemic. A *p*-value < 0.05 was considered to indicate statistical significance.

## 3. Results

This study sample included 42.6% male and 57.4% female participants. The age distribution primarily comprised individuals aged 61–80 years (58.8%), followed by those aged 40–60 years (38.5%), with a smaller portion aged 81–100 years (2.6%). The ethnic/racial breakdown included 73.5% White, 13.9% Black, 7.0% Asian, and 4.3% Hispanic participants. In terms of education, 51.6% held a college degree or higher, 31.9% had a college education, and 16.3% had less than a high school education. Regarding annual income, 37.7% reported earning less than USD 50,000, 32.7% earned between USD 50,000 and USD 99,999, and 25.5% earned more than USD 100,000 (Table 1).

Based on the paired-samples *t*-tests, the mean DST scores for the consumption of fruits (female: mean percentage change (MPC): −4.71%; male: MPC = −3.58%), grains (female: MPC = −8.24%; male: MPC = −6.43%), lean protein (female: MPC = −1.35%; male: MPC = −1.47%), and dairy (female: MPC = −1.34%; male: MPC = −1.44%) significantly decreased for both sexes after the pandemic began. However, the mean FSS consumption score significantly increased in both men (3.96%) and women (3.41%), indicating healthier habits or decreased FSS consumption. Men showed a significant increase in vegetable consumption (0.93%), whereas there was no significant change in vegetable consumption among women after the onset of the COVID-19 pandemic. The only food group consumption status that did not significantly differ between the two sexes after the onset of the pandemic was processed meat. The largest reduction in consumption was associated with grain consumption by women (8.2%) compared with men (6.4%).

Based on the chi-square test, there were several sex differences in changes in food consumption during the COVID-19 pandemic. Compared with women, men reported increased consumption of four food groups: fruit (male = 19.5% vs. female = 17%), vegetables (male = 20.7% vs. female = 18.9%), dairy (male = 16% vs. female = 14.6%), and processed meat (male = 16.5% vs. female = 15.6%). The most significant decrease in consumption after the onset of the COVID-19 pandemic was observed in FSS intake by both men and women (39.9%), although there was no significant difference between the sexes. In contrast, the most considerable increase in consumption after the onset of the COVID-19 pandemic was related to FSS intake, with an increase of 23.6% in females and 22.7% in males. Table 2 provides the details of the findings.

The mean scores for fruit and grain consumption significantly decreased in all three age groups compared with those before and during the pandemic. However, the mean FSS consumption score significantly increased across all the age groups during this period. There was a significant decrease in the mean lean protein consumption score of 2.6% for those aged 40–60 years, whereas there was no significant change in the other two age groups. In addition, there were significant decreases in the mean dairy consumption score of 2.1% and 0.8% in the 40–60- and 61–80-year age groups, respectively. The mean score for the consumption of processed meat significantly decreased only for the 61–80-year age group. Interestingly, vegetable consumption significantly decreased by 1.6% among participants aged 40–60 years, whereas it significantly increased by 1.3% and 4.3% among those aged 61–80 and 81–100 years, respectively. The largest percentage change before and during the pandemic was observed for grain consumption, with significant decreases of 7.9% and 6.2% among those aged 61–80 and 81–100 years, respectively (Table 2).

Subsequent analysis revealed the distribution of different food consumptions across the three age groups, namely, “decreased consumption”, “no change”, and “increased consumption”. These results suggested a statistically significant association between age and food consumption.

After the onset of the COVID-19 pandemic, decreased consumption of fruits (“40–60” = 33.6% vs. “61–80” = 29.9% vs. “81–100” = 27.4%), grains (“40–60” = 32.7% vs. “61–80” = 31.9% vs. “81–100” = 30%), vegetables (“40–60” = 22.4% vs. “61–80” = 15.2% vs. “81–100” = 12.5%), and processed meat (“40–60” = 18.3% vs. “61–80” = 12.5% vs. “81–100” = 9.5%) was observed across age groups, with lower food consumption rates observed in older age groups. The greatest decrease in lean protein consumption was observed in the “40–60” age group (21%), while in the “61–80” and “81–100” age groups, the reduction in lean protein consumption was approximately 15.5%. In addition, the reductions in dairy consumption were as follows: 19.9% in the “61–80” age group, 17.9% in the “81–100” age group, and 14% in the “61–80” age group. The reduction in FSS was greater in comparison with that in the other food groups for all age groups. The “40–60” age group had a decrease in FSS consumption of 41.5%, while the “81–100” and “61–80” age groups had decreases of 41.1% and 38.1%, respectively, after the onset of the COVID-19 pandemic. In contrast, the greatest increase in consumption after the onset of the COVID-19 pandemic was associated with vegetable intake, with an increase of 25.5% in the “81–100” age group, followed by an increase in the FSS of 23.9% in the “40–61” age group (Table 3).

The results of the binary logistic regression model are reported in Table 4. The results revealed that sex (reference: female) was a significant independent predictor of lower consumption of dairy and processed meat after the onset of the COVID-19 pandemic. Males were approximately 30% more likely to reduce their consumption of dairy and processed meat after the onset of the COVID-19 pandemic than females (OR = 1.31, 95% confidence interval (CI) = 1.16–1.47, *p* < 0.0001 for dairy) (OR = 1.29, 95% CI = 1.16–1.44, *p* < 0.0001 for processed meat).

Age was a significant predictor of reduced fruit (*p* = 0.031), vegetable (*p* < 0.0001), lean protein (*p* < 0.0001), dairy (*p* < 0.0001), and processed meat (*p* < 0.0001) consumption after the onset of the COVID-19 pandemic. However, the results suggested that age and sex were not independent predictors of changes in grain consumption or FSS after the onset of the pandemic (*p* > 0.05). The following report lists the changes in food consumption due to COVID-19.

Processed meats: The “40–60” age group was 130.9% more likely to reduce processed meat consumption than the “81–100” age group (OR = 2.309, 95% CI: 1.444–3.692, *p* < 0.0001). There was no significant difference in processed meat consumption between the “61–80” and “81–100” age groups.

Vegetables: The likelihood of decreasing vegetable consumption in the “40–60” and “61–80” age groups was 96% and 25%, respectively. The difference was statistically significant for “40–60” vs. “81–100” (OR = 1.96, 95% CI = 1.32–2.91, *p* < 0.0001) but not for “61–80” vs. “81–100”. Additionally, compared with the “61–80” age group, the “40–60” age group was 57% more likely to reduce vegetable consumption after the onset of the COVID-19 pandemic.

Lean protein: The “40–60” age group was 33% more likely to reduce lean protein consumption than the “81–100” age group (OR = 1.33, 95% CI = 0.92–1.91, *p* = 0.0037). No significant differences were observed between the other age groups.

Dairy: the “40–60” age group was 11% more likely to reduce dairy consumption after the onset of the COVID-19 pandemic compared with the “81–100” age group (OR = 1.11, 95% CI = 0.78–1.57, *p* = 0.0239), while the “61–80” age group was 21% less likely to decrease dairy consumption than the “81–100” age group (OR = 0.784, 95% CI = 0.55–1.108, *p* = 0.0022).

Fruit: The “40–60” and “61–80” age groups were 18% and 5% more likely to have decreased fruit consumption, respectively, than the “81–100” age group after the onset of the COVID-19 pandemic. However, the CIs for both OR estimates include 1, indicating that the difference in the probability of decreased consumption of fruit after the onset of the COVID-19 pandemic between these age groups and the reference category (“81–100”) is not statistically significant at the 95% confidence level; in other words, the differences in odds were not sufficiently large to be considered clinically significant, and the OR estimates suggest that these differences may be due to chance.

The percentages of females and males in the “not at risk” group were approximately 7% and 6.7%, respectively, before the pandemic. After the onset of the COVID-19 pandemic, the percentage of females in this group decreased to 6.59%, whereas the percentage of males increased to 7.05%. In the “possible risk” group, before COVID-19, 37.25% of the participants were females and 35.79% were males; however, after the onset of the pandemic, these numbers decreased to 35.06% for females and 34.09% for males. Finally, in the “at risk” group, before COVID-19, 55.78% of the participants were females and 57.53% were males, and after the onset of the pandemic, these percentages increased to 58.35% for females and 58.86% for males.

Table 5 shows the distribution of participants in the three age groups and their sex based on their nutritional risk status before and after the onset of the pandemic. Risk status was categorized into three groups: “not at risk”, “possible risk”, and “at risk”. Before the pandemic, the percentage of participants in the “not at risk” category was highest in the “81–100” age group (8.33%) and lowest in the “40–60” age group (5.43%). After the onset of the pandemic, the percentage of participants in this category very slightly increased in the “40–60” age group and by 3% in the “81–100” age group.

For the “possible risk” category, 45.8% of individuals were in the “81–100” age group before the onset of the pandemic, followed by the “61–80” and “40–60” age groups (37.5% and 34.64%, respectively). After the onset of the pandemic, the percentage of individuals classified as at “possible risk” decreased in all age groups, with the greatest decrease observed in the “81–100” age group (4.9%).

Before the pandemic, the percentage of participants in the “at risk” category was highest in the “40–60” age group (59.9%) and lowest in the “81–100” age group (45.8%). After the onset of the pandemic, the percentage of participants in this category increased in all age groups, with the highest increase of 2.1% observed in the “61–80” age group.

Table 6 shows the mean (SD) percentage change (MPC) in DST scores before and after the pandemic, as well as the *p*-values for the comparisons between the two time points stratified by sex and age. The mean percentage change before and after the onset of the COVID-19 pandemic was 1.78% for females and 1.76% for males, indicating that both male and female participants experienced a statistically significant decline in their nutritional risk status after the onset of the COVID-19 pandemic.

The MPC was 1.78% and 1.76% for the “40–60” and “61–80” age groups, respectively, indicating a slight overall improvement in nutritional risk status after the onset of the COVID-19 pandemic. The *p*-value for both age groups was <0.001, suggesting that the change was statistically significant. However, the MPC for the “81–100” age group was only 1.73%, which was slightly lower than that of the other age groups, and the *p*-value was not statistically significant (0.660), indicating that there was no significant change in nutritional risk in this age group after the onset of the COVID-19 pandemic.

The DST scores of the participants in the “40–60” and “61–80” age groups significantly decreased after the onset of the pandemic, with MPCs of 1.78% and 1.76% for the “40–60” and “61–80” age groups, respectively. However, the nutritional risk of the “81–100” age group did not significantly change after the onset of the COVID-19 pandemic.

Subsequent binary logistic regression analysis revealed that, based on nutritional risk, female sex was significantly associated with a higher probability of being classified as “nutritionally vulnerable” after the onset of the COVID-19 pandemic (OR = 1.17, 95% CI = 1.01–1.36, *p* = 0.0310). The OR for sex showed that compared with males, females had 17.3% greater odds of being classified as “nutritionally vulnerable” after the onset of the COVID-19 pandemic after controlling for the other variables in the model.

The results also showed that age had no statistically significant effect on the probability of being classified as “nutritionally vulnerable” after the onset of the COVID-19 pandemic. The ORs for age groups compared with the reference category (“81–100”) are both close to 1 and have wide CIs that include 1, indicating that the differences in the probability of being classified as “nutritionally vulnerable” among the age groups are not statistically significant (OR = 1.51, 95% CI = 0.88–2.598, *p* = 0.1126 for “40–60” vs. “81–100”) (OR = 1.43, 95% CI = 0.84–2.43, *p* = 0.2964 for “61–80” vs. “81–100”).

## 4. Discussion

The COVID-19 pandemic has profoundly affected many aspects of daily life, including dietary habits and nutritional intake. This study aimed to assess the changes in dietary habits among adults of different sex and age groups during the latter stage of the COVID-19 outbreak. Uniquely, we investigated (1) how consumption of MyPlate food items in conjunction with the FSS changed after the onset of the pandemic, (2) whether these changes differed by sex and age, and (3) whether sex and age predicted the likelihood of becoming nutritionally vulnerable. The findings of this study are important because they provide valuable insights regarding individuals who are at greater risk of pandemic-related nutritional variation. Furthermore, the findings highlight the food groups that require more attention during future health crises to avoid harm to vulnerable populations.

Sex differences played a role in shaping dietary changes during the COVID-19 pandemic. Based on the results of a binary logistic regression analysis, sex was identified as a significant predictor of a decrease in dairy and processed meat consumption during the COVID-19 pandemic among the different food groups studied. Specifically, males were 30% more likely to reduce their consumption of these food groups than females. A recent study showed that a moderate intake of dairy products may reduce the risk of COVID-19 by 37%, whereas a higher intake of low-fat dairy products may provide additional protection against the disease [46]. This implies that while the benefits of dairy consumption during the COVID-19 pandemic are noteworthy, it is unknown whether sex differences have affected dairy consumption after the onset of the COVID-19 pandemic. Furthermore, despite a recent study showing that processed meat consumption did not significantly change during the COVID-19 pandemic [45], when we divided the data by sex, we found that processed meat consumption increased more among females than males during the pandemic. This could be due to the affordability and availability of processed meat, as women are more likely to experience a negative change in economic status [24,25,31]. However, further research is required to better understand the reasons for these sex-related differences in food consumption.

The COVID-19 pandemic resulted in a significant reduction in the consumption of various food groups, and this trend appeared to vary across age groups. This study found that, compared with individuals aged 81–100 years, those aged 40–60 years reported greater reductions in the consumption of processed meat (131%), vegetables (96%), lean protein (33%), and dairy (11%). These findings suggest that older adults may have maintained healthier dietary patterns during the pandemic, possibly due to increased health awareness and more cautious behavior in response to their heightened vulnerability to COVID-19. Given the elevated risk of severe illness and complications among older adults, they may have been more motivated to make health-promoting dietary choices to strengthen their immune systems and overall resilience. Additionally, this study revealed that individuals in the 61–80 age group were 21% less likely to decrease dairy consumption compared to those in the 81–100 age group. This may reflect a higher awareness in this age group of the role of dairy in maintaining bone health and preventing age-related conditions such as osteoporosis. Overall, these findings underscore the importance of understanding age-related differences in dietary behavior during public health crises and tailoring nutritional guidance accordingly.

In addition, the results suggest that female sex may be associated with a greater probability of being “nutritionally vulnerable” after the onset of the COVID-19 pandemic, and age is not a strong predictor of nutritional vulnerability. There may be sex differences in the impact of the pandemic on food security and access to healthy foods. According to recent research findings, compared with men, women tend to consume more food because of fear, anxiety, or boredom; prefer to eat more unhealthy foods; stockpile a greater amount of food; and frequently modify their shopping habits. Women are more likely to experience job loss or reduced working hours due to the pandemic, which could affect their ability to afford healthy foods [25,47,48,49]. Women are also more likely to be the primary caregivers of children or elderly relatives [50], which could limit the time and resources available for them to obtain and prepare healthy meals. Thus, the changes in daily routines resulting from quarantine had a greater impact not only on women but also on the entire family, as women typically have more responsibility for making decisions about family food choices [20]. The age groups that were not significantly associated with nutritional vulnerability may not be directly associated with the changes in food access, affordability, or food-related behaviors that may have led to nutritional vulnerability during the pandemic.

One major strength of this study was its large sample size, which enhanced the statistical power and precision of the results. While a large sample size does not automatically guarantee representativeness, the diverse demographic characteristics of the sample improve the generalizability of the findings. Additionally, the large sample size makes the ORs more dependable and reduces the likelihood that significant results are due to chance. However, we acknowledge that selection bias may still be a potential limitation despite this study’s size. The other strengths of this study include the consideration of all MyPlate food items in conjunction with the FSS consumption score, which provided a comprehensive assessment of dietary changes during the COVID-19 pandemic. The inclusion of data from diverse age groups and both sexes in this study provided more detailed insights into the variations in dietary habits that exist among different populations. Additionally, the findings provide valuable insights into the groups of individuals who are at greater risk of nutritional changes due to the COVID-19 pandemic, thus highlighting food groups that require more attention during future crises to avoid harm to vulnerable individuals. One limitation of this study was the use of self-reported data, which may be subject to recall and social desirability biases. The participants may have overreported their consumption of healthy foods and underreported their consumption of unhealthy foods, leading to an inaccurate portrayal of their dietary habits during the pandemic. Additionally, participants may have had difficulty accurately recalling their pre-pandemic dietary habits, making it challenging to compare changes in consumption patterns. Additionally, this study focused on changes in dietary habits during the later stages of the pandemic, and it is unclear how these changes may have evolved over time or how they may continue to change as the pandemic persists. Additionally, determinants of differences in food intake behavior, such as participants’ COVID-19 infection status or their social environment during the pandemic, could not be identified in this study. Future research should explore these potential influences to better understand the drivers of dietary changes during the pandemic. Finally, this study did not assess the long-term health implications of changes in dietary habits during the pandemic. Further research is needed to understand the potential consequences of these changes on health outcomes. Although age groups were categorized to reflect middle-aged, early senior, and advanced senior populations, we recognize that individuals within the ages of 61–80 may differ in lifestyle from older adults in the same group. Future studies could use narrower age ranges to better capture such differences.

### Implications for Research and Practice

Although this study provides valuable insights into the changes in food consumption after the onset of the COVID-19 pandemic and how they vary by age and sex, there are several implications for future studies. A longitudinal study design can be used to track changes in dietary behavior over time, providing a more accurate understanding of nutritional patterns. More in-depth qualitative methods, including interviews and focus groups, should be employed to explore the reasons for these dietary changes. Cultural and socioeconomic differences in food consumption changes during pandemics can be studied to tailor interventions to specific populations. Future studies should examine the impact of changes in food consumption on health outcomes and explore the effectiveness of different behavioral interventions in promoting healthy food choices during pandemics.

## 5. Conclusions

This study highlighted the effects of the COVID-19 pandemic on lifestyle behaviors, particularly diet, across various age groups and in both sexes. This study examined changes in the consumption patterns of seven different food items, including MyPlate food items (with separate categories for lean protein and processed meat), as well as the FSS consumption score. In this study, we aimed to identify groups that were at greater risk of experiencing nutritional changes due to the COVID-19 pandemic. These findings suggested that sex plays a role in shaping dietary changes. Female sex was associated with a greater probability of nutritional vulnerability after the onset of the COVID-19 pandemic. Moreover, older adults had healthier dietary patterns than younger adults during the pandemic. However, age was not a significant predictor of being classified as “nutritionally vulnerable” after the onset of the COVID-19 pandemic. Overall, this study underscores the need for tailored dietary advice that considers the unique challenges faced by different age groups and both sexes during the pandemic.

## Figures and Tables

**Table 1:** Demographic characteristics of the participants.

Characteristic	Frequency (*N*)	Percentage (%)
**Sex**		
Male	4283	42.62
Female	5767	57.38
**Age (years)**		
40–60	3866	38.47
61–80	5908	58.79
81–100	263	2.62
Missing	13	0.13
**Ethnicity/Race**		
White	7390	73.53
Black	1393	13.86
Asian	701	6.98
Hispanic	429	4.27
Missing	137	1.36
**Education**		
Less than high school	1634	16.26
Some college	3210	31.94
College degree and higher	5191	51.65
Missing	15	0.15
**Annual income (USD)**		
Less than 25,000	1551	15.43
25,000–49,999	2243	22.32
50,000–99,999	3281	32.65
100,000 or more	2566	25.53
Missing	409	4.07

**Table 2:** Scores and consumption of food items after the onset of the COVID-19 pandemic based on sex.

Food Items	Sex	Before	After	MPC ^1^	*p*-Value ^2^	Decreased Consumption	No Change	Increased Consumption	*p*-Value ^3^
		Mean (SD)	Mean (SD)	(%)		N (%)	N (%)	N (%)	
Fruits	Female	8.3 (3.58)	7.9 (3.73)	−4.71	* <0.001	1828 (31.70)	2960 (51.33)	979 (16.98)	
	Male	8.75 (3.83)	8.44 (3.97)	−3.58	* <0.001	1316 (30.73)	2131 (49.75)	836 (19.52)	
	*p*-value								* 0.0047
Grains	Female	7.13 (4.49)	6.54 (4.55)	−8.24	* <0.001	1892 (32.81)	2986 (51.78)	889 (15.42)	
	Male	8.01 (4.51)	7.49 (4.56)	−6.43	* <0.001	1339 (31.26)	2264 (52.86)	680 (15.88)	
	*p*-value								0.2580
Vegetables	Female	8.49 (4.09)	8.48 (4.21)	−0.20	0.88	1023 (17.74)	3655 (63.38)	1089 (18.88)	
	Male	8.03 (4.15)	8.1 (4.22)	0.93	* 0.01	780 (18.21)	2616 (61.08)	887 (20.71)	
	*p*-value								* 0.0377
Lean protein	Female	5.17 (2.49)	5.1 (2.54)	−1.35	* 0.003	1018 (17.65)	3914 (67.87)	835 (14.48)	
	Male	5.27 (2.4)	5.19 (2.5)	−1.47	* 0.008	757 (17.67)	2855 (66.66)	671 (15.67)	
	*p*-value								0.2407
Dairy	Female	4.4 (2.47)	4.34 (2.49)	−1.34	* 0.001	883 (15.31)	4044 (70.12)	840 (14.57)	
	Male	4.64 (2.7)	4.57 (2.74)	−1.44	* 0.003	763 (17.81)	2836 (66.22)	684 (15.97)	
	*p*-value								* 0.0001
Fat, sugar, and sweets	Female	13.89 (4.3)	14.37 (4.52)	3.41	* <0.001	2260 (39.90)	2144 (37.18)	1363 (23.63)	
	Male	13.37 (4.66)	13.9 (4.86)	3.96	* <0.001	1708 (39.88)	1604 (37.45)	971 (22.67)	
	*p*-value								0.5148
Processed meats	Female	7.56 (2.58)	7.52 (2.56)	−0.548	0.07	788 (13.66)	4077 (70.70)	902 (15.64)	
	Male	6.7 (2.76)	6.71 (2.7)	0.15	0.93	682 (15.92)	2895 (67.59)	706 (16.48)	
	*p*-value								* 0.0014

^1^. Mean percentage change; *. Indicates a significant relationship at *p* < 0.05; ^2^. Paired-samples *t*-test; ^3^. Chi-square test.

**Table 3:** Scores and consumption of food items after the onset of the COVID-19 pandemic based on age.

Food Items	Age Range (Years)	Before	After	MPC ^1^	*p*-Value ^2^	Decreased Consumption	No Change	Increased Consumption	*p*-Value ^3^
		Mean (SD)	Mean (SD)	(%)		N (%)	N (%)	N (%)	
Fruits	40–60	8.39 (3.82)	7.98 (3.97)	−4.93	<0.001	1299 (33.60)	1757 (45.45)	810 (20.95)	
	61–80	8.51 (3.62)	8.18 (3.75)	−3.79	<0.001	1767 (29.91)	3175 (53.74)	966 (16.35)	
	81–100	9.5 (3.66)	9.16 (3.83)	−3.60	<0.001	72 (27.38)	155 (58.94)	36 (13.96)	
	*p*-value								* <0.0001
Grains	40–60	7.33 (4.41)	6.84 (4.43)	−4.93	<0.001	1264 (32.70)	1856 (48.01)	746 (19.30)	
	61–80	7.57 (4.57)	6.98 (4.65)	−7.89	<0.001	1883 (31.87)	3240 (54.84)	785 (13.29)	
	81–100	8.37 (4.71)	7.84 (4.91)	−6.23	<0.001	79 (30.04)	150 (57.03)	34 (12.93)	
	*p*-value								* <0.0001
Vegetables	40–60	8.29 (4.2)	8.15 (4.33)	−1.63	0.02	867 (22.43)	2200 (56.91)	799 (20.67)	
	61–80	8.29 (4.07)	8.4 (4.15)	1.33	<0.001	899 (15.22)	3903 (66.06)	1106 (18.72)	
	81–100	8.57 (4.04)	8.93 (4.06)	4.26	0.009	33 (12.55)	163 (61.98)	67 (25.48)	
	*p*-value								* <0.0001
Lean protein	40–60	5.21 (2.45)	5.08 (2.55)	−2.63	<0.001	811 (20.98)	2390 (61.82)	665 (17.20)	
	61–80	5.2 (2.45)	5.16 (2.49)	−0.78	0.12	918 (15.54)	4201 (71.11)	789 (13.35)	
	81–100	5.42 (2.41)	5.56 (2.6)	2.45	0.13	41 (15.59)	173 (65.78)	49 (18.63)	
	*p*-value								* <0.0001
Dairy	40–60	4.74 (2.58)	4.63 (2.61)	−2.15	<0.001	769 (19.89)	2403 (62.16)	694 (17.95)	
	61–80	4.35 (2.56)	4.32 (2.58)	−0.78	0.03	826 (13.98)	4284 (72.51)	798 (13.51)	
	81–100	5.42 (2.41)	5.56 (2.6)	2.45	0.09	47 (17.87)	184 (69.96)	32 (12.17)	
	*p*-value								* <0.0001
Fat, sugar, and sweets	40–60	13.46 (4.8)	14.08 (4.98)	4.61	<0.001	1603 (41.46)	1340 (34.66)	923 (23.87)	
	61–80	13.84 (4.22)	14.26 (4.48)	2.97	<0.001	2250 (38.08)	2300 (38.93)	1358 (22.99)	
	81–100	12.9 (4.55)	13.59 (4.64)	5.07	<0.001	108 (41.06)	105 (39.92)	50 (19.01)	
	*p*-value								* 0.0002
Processed meats	40–60	6.67 (2.89)	6.69 (2.8)	0.43	0.53	707 (18.29)	2456 (63.53)	703 (18.18)	
	61–80	7.25 (2.5)	7.47 (2.5)	−0.64	0.02	736 (12.46)	4306 (72.88)	866 (14.66)	
	81–100	7.81 (2.52)	7.73 (2.55)	−1.02	0.52	25 (9.51)	203 (77.19)	35 (13.31)	
	*p*-value								* <0.0001

^1^. Mean percentage change. ^2^. Paired samples *t*-test; ^3^. Chi-square test. *. Indicates a significant relationship at *p* < 0.05.

**Table 4:** Consumption of food items based on age and sex while controlling for confounding variables.

	Reference	Odds Ratio	95% CI ^1^	*p*-Value ^2^
Fruits				
Sex	Female	0.995	0.909–1.090	0.9174
Age				* 0.0308
	40–60 vs. 61–80	1.132	1.030–1.244	
	40–60 vs. 81–100	1.179	0.881–1.577	0.0823
	61–80 vs. 81–100	1.041	0.782–1.387	0.5996
Grains				
Sex	Female	1.043	0.954–1.142	0.3548
Age				0.7541
	40–60 vs. 61–80	0.975	0.889–1.073	
	40–60 vs. 81–100	1.067	0.799–1.425	0.8031
	61–80 vs. 81–100	1.093	0.823–1.452	0.4741
Vegetables				
Sex	Female	0.949		0.3533
Age				* <0.0001
	40–60 vs. 61–80	1.571	1.404–1.758	
	40–60 vs. 81–100	1.968	1.327–2.918	* <0.0001
	61–80 vs. 81–100	1.253	0.848–1.851	0.2892
Lean protein				
Sex	Female	0.929	0.832–1.038	0.1947
Age				* <0.0001
	40–60 vs. 61–80	1.362	1.215–1.526	
	40–60 vs. 81–100	1.331	0.926–1.915	* 0.0037
	61–80 vs. 81–100	0.978	0.683–1.400	0.0952
Dairy				
Sex	Female	0.771	0.689–0.864	* <0.0001
Age				* <0.0001
	40–60 vs. 61–80	1.416	1.260–1.592	
	40–60 vs. 81–100	1.110	0.782–1.577	* 0.0239
	61–80 vs. 81–100	0.784	0.555–1.108	* 0.0022
Fat, sugar, and sweets				
Sex	Female	0.926	0.850–1.009	0.0793
Age				
	40–60 vs. 61–80	1.089	0.995–1.191	
	40–60 vs. 81–100	0.937	0.717–1.226	0.8965
	61–80 vs. 81–100	0.860	0.661–1.120	0.1092
Processed meats				
Sex	Female	0.764	0.679–0.861	* <0.0001
Age				* <0.0001
	40–60 vs. 61–80	1.470	1.302–1.661	
	40–60 vs. 81–100	2.309	1.444–3.692	* <0.0001
	61–80 vs. 81–100	1.570	0.986–2.501	0.7936

^1^. Confidence interval; ^2^. Binary logistic regression models with MyPlate food items as the response variable and predictor variables, including sex, race, age, income, and education. *. Indicates a significant relationship at *p* < 0.05.

**Table 5:** Nutritional risks based on age group and sex before and after the onset of COVID-19.

		Female	Male	40–60	61–80	81–100
		N (%)	N (%)	N (%)	N (%)	N (%)
Not at risk	Before	402 (6.97)	286 (6.68)	210 (5.43)	455 (7.70)	22 (8.33)
	After	380 (6.59)	302 (7.05)	211 (5.46)	440 (7.45)	30 (11.36)
Possible risk	Before	2148 (37.25)	1533 (35.79)	1339 (34.64)	2215 (37.49)	121 (45.83)
	After	2022 (35.06)	1460 (34.09)	1267 (32.77)	2103 (35.59)	108 (40.91)
At risk	Before	3217 (55.78)	2464 (57.53)	2317 (59.93)	3239 (54.81)	121 (45.83)
	After	3365 (58.35)	2521 (58.86)	2388 (61.77)	3366 (56.96)	126 (47.73)

**Table 6:** DST scores before and after the onset of the COVID-19 pandemic based on age and sex.

	Before	After	MPC	*p*-Value
	Mean (SD)	Mean (SD)	(%)	
Sex				
Female	56.13 (0.16)	55.49 (0.16)	1.78	* <0.001
Male	55.91 (0.19)	55.59 (0.19)	1.76	* <0.001
Age (years)				
40–60	55.43 (0.19)	54.85 (0.20)	1.78	* <0.001
61–80	56.35 (0.16)	55.87 (0.16)	1.76	* <0.001
81–100	57.96 (0.75)	58.10 (0.78)	1.73	0.660

DST: Dietary Screening Tool. *. Indicates a significant relationship at *p* < 0.05.

## Data Availability

Data used during the current study are available from the corresponding author. The data is not shared publicly due to be a part of an ongoing study.

## Abbreviations

CFR	Case fatality ratio
DST	Dietary Screening Tool
(FSS)	Fat, sugar, and sweet
GTFN	Gender-transformative framework for nutrition
MPC	Mean percentage change
LCP	Life course perspective
